# The Big Flush of Montreal: On affective maintenance and infrastructural events

**DOI:** 10.1177/03063127221126465

**Published:** 2022-10-14

**Authors:** Kregg Hetherington, Elie Jalbert

**Affiliations:** Concordia University, Montreal, QC, Canada

**Keywords:** infrastructure, publics, eventfulness, temporality, maintenance, expertise, affect

## Abstract

This article is about a brief controversy that erupted in 2015 around the City of Montreal’s plan to divert 8 billion liters of raw sewage into the St. Lawrence River while it conducted critical maintenance on its sewer infrastructure. In the end, though, the Flush was non-eventful: It went ahead as planned and with no lasting effects or complaints. We suggest that the best way to understand how the City averted the crisis is through the concept of ‘affective maintenance’. If infrastructures are meant to be uneventful (i.e. narratively stable and generally lacking in surprise ruptures) then the maintenance of public affect is as important to their functioning as the physical work that keeps sewage flowing in the right direction.

In mid-September 2015, Montreal City Hall announced the schedule of public works projects planned for the month of October. Among these plans was maintenance work on the south-east interceptor, a massive sewer-pipe whose existence rarely received public attention. Installed in the 1980s, the interceptor system rings most of the island of Montreal, gathering sewage from smaller pipes and sending it to the filtration station at the island’s eastern extremity. Reinforcements inside the pipe had begun to rust and break, threatening to damage the filtration station. The City saw a window of opportunity for these renovations during the rebuilding of a major autoroute and a snow dump, both of which required closing the interceptor. By doing all three maintenance jobs at the same time, the City would minimize the number of times the interceptor would be closed.

Posting these plans was a routine gesture, required by City bylaws. The response to the announcement, however, was anything but routine. The opposition party, Projet Montréal, sounded the alarm on September 28th that, with the closure of the interceptor, 8 billion litres of raw sewage would be piped directly into the river at some 20 different outflows (CBC, 2015b). A public outcry ensued, and by the next day, the press had termed the imminent event ‘Flushgate’, and later ‘The Big Flush’. A cross-section of Montrealers protested in the streets and on social media, and the controversy soon involved nearby municipalities, Indigenous communities, the provincial and federal governments, and even international politicians and journalists. What the administration had presented as cooperative, proactive maintenance, opponents now took as a sign of incompetence, malfeasance, or environmental negligence. For a brief moment, the looming Flush promised to be a major event, a rupture in Montrealers’ daily lives that might fundamentally change the way wastewater was managed in the City.

And then, very quickly, the Big Flush subsided. After a slight delay, the work went forward as planned. Raw sewage was indeed flushed into the river, but, as the City had promised, no perceptible damage was done to the river, or rather any damage to the river slipped below the threshold of attention, and so didn’t have lasting consequences on political careers or infrastructural design. The Big Flush was a non-event, a blip of administrative history without serious consequences. But the fact that Montrealers remember the Big Flush, that it got its own proper noun, suggests that it did almost produce such a shift. Indeed, if most commentators on the Flush were relieved that it hadn’t amounted to much, many did see it as something of a missed opportunity, a political configuration that perhaps *should have been* eventful.

In this paper we suggest that non-events, or almost-events like this one, have much to tell us about infrastructural temporality. The invention of modern sewers in the 19th century is memorialized as one of the foundational events in the constitution of urban governance as we know it today (Gandy, 2014): an intervention into the flow of water and waste that promised a whole new kind of public life. Today, however, the ideal for such sewers is to remain as uneventful as possible, such that they fall away from public consciousness almost entirely. When sewers become visible it is because something has gone wrong, a flood or a breakdown, or a body out of place. Maintenance on sewers is not, therefore, just about ensuring that they keep working, but also that they remain invisible, that they fail to draw attention.

The work of maintenance, in this sense, is affective and political. As Knox (2017, p. 375) puts it, ‘politics … is neither prior to nor determined by material structures, but emerges and is reworked through affective engagements with the material arrangements of the worlds in which people live’. Non-events like the Big Flush are as much produced by the careful management of perception as they are by well-planned technical interventions. We call that work ‘affective maintenance’ here, to underline the careful work that goes into managing infrastructural temporality and the complex relations that hold an infrastructure and its public in place. Based on a review of newspaper articles, government websites, and interviews with Montreal-based sewage experts,^1^ this paper explores two kinds of affective maintenance that helped render the Big Flush non-eventful. The first is populist: As the Big Flush threatened to create a new, disgruntled public, the mayor’s office sought to articulate that public differently, formulating a communal urban subject that saw the event not as a disgusting surprise but as a matter of communal responsibility. The second was the appeal to expertise, which deferred the crisis by temporarily removing its affective charge. Had the Big Flush erupted into the scandal that, for a brief period, it seemed it might, Montreal might have found itself seriously reconsidering the way that it dealt with its waste, changing its politicians, its infrastructure, and its relation to the river that surrounds it. But instead, deft affective maintenance allowed the process to go ahead uneventfully, and for the pipes, waters, and municipal bureaucracy to recede once more from view.

## Sewage, infrastructure, and event

Well-functioning infrastructure is, almost by definition, non-eventful. In Larkin’s (2013, p. 328) fortuitous phrasing, ‘infrastructures are built structures that facilitate the flow’ of other things; in a world of things in motion, in other words, infrastructures offer constants. There’s a poetics, of course, to the building of infrastructures, an affective charge to their inauguration, in which their appearance on a landscape promises some sort of future, and this is why infrastructural building and thinking was so key to 20th-century notions of progress and development (Harvey, 2018; Knox, 2017). These events, as Wagner-Pacifici (2017) puts it, are narrative cleavages that signal a before and an after, and thereby help to establish the directionality of progressive time (see also Gupta, 2018; Hetherington, 2017). And for the same reason, after the ribbon-cutting, most people do not want their bridges to be eventful, nor their sewers. A functioning infrastructure is precisely one that admits no temporal cleavages. This is a key part of what maintenance does: preventing infrastructure from becoming eventful by keeping a particular set of relationships functioning in more or less the same way.^2^

This temporal argument is a slight reframing of one of the classic axioms of infrastructure studies: that infrastructure becomes visible only upon breakdown (Star & Ruhleder, 1996). As we’ll see, the reverse can also be true: making infrastructure visible can lead to a kind of breakdown as well. The perception that an infrastructure is not working, or a shift in expectation about what an infrastructure ought to do, can be just as consequential as a strictly material malfunction. That fact brings attention to the affective dimensions of infrastructure, about the way in which people’s perceptions and feelings about roads and pipes are densely connected to how they work (Knox, 2017). And that suggests that in addition to the kind of material maintenance that goes into the smooth functioning of something like a sewer pipe, there is often a kind of ‘affective maintenance’ at work as well,^3^ a tinkering with how a sewer’s publics sense the pipe itself, it’s importance to their lives, or the significance of any changes that might occur around it. By extension, an infrastructural event is not just a breakdown but a shuddering reconfiguration of material practices and affective dispositions, which may as easily create new publics and expectations as send water off in a new direction. Infrastructural invisibility is itself a site of struggle, where different narrators compete over alternative stories of development. Even marginal differences in interpretation can lead some to argue that emergent infrastructural configurations are exceptional, unstable, or dangerous, against others who claim that they are merely normal variations that do not meaningfully change what people have grown used to.

The kind of affective maintenance we are describing here is best thought of as part of a cosmopolitics of infrastructure. For Stengers (2010), cosmopolitics refers to those contests over the material arrangement of the world in which different actors advance ‘propositions’ about those arrangements. In a cosmopolitical sense, the question of whether the Big Flush was an environmental calamity or an act of routine maintenance cannot be fully resolved by appeals to what *is*. Rather, they are competing propositions that themselves enter the fray, affecting the very relationships they propose to describe. Part of the work that such propositions do in the world is affective: They compel attention or dispel it, gathering actors around particular material arrangements. Infrastructural events are thus moments of dramatic affective shifts that interrupt narratives of progress or stability. Whole publics that depend on those narratives (wittingly or not) come to see those arrangements differently.

Take one of the best-known events in the history of sewage, the Great Stink of London, largely responsible for the invention of the modern interceptor system that Montreal uses today. As Halliday (1999) tells this famous story, it was the summer of 1858, when a heat wave made the stench of the river Thames unbearable to anyone unfortunate enough to live or work nearby. The problem had been building for decades. In the early 1800s, London’s sewers were only intended to drain storm water, which they collected from the massive network of roads and dumped, at hundreds of different points, directly into the Thames, which flowed through the middle of the city. Household waste was still, at that point, a private matter, with households collecting ‘night soil’ in their yards and basements, then paying collectors to gather it up and sell it as fertilizer to farms outside of London. Floods, in other words, were a public affair, while shit was not. But in the 1830s, when wealthier households began to install newly invented ‘water closets’ to reduce the sanitation problems in their own homes, they connected these directly to the municipal storm drains. The rapid adoption of the WC was seen as a sign of progress, until the 1840s, when the impact on the Thames became apparent: First, fish began to disappear, and soon rare diseases such as dysentery and typhoid began to wreak havoc on poorer communities living near the river. When cholera first appeared in London, engineers and public health experts called for a complete reform of the sewage system. A total of six royal commissions were carried out, and the greatest engineers of the day proposed different solutions, including building giant interceptor pipes along both sides of the Thames that would cut off the sewers and carry the waste downstream away from the city.

According to Halliday, however, neither the disease nor the stench sufficiently affected the political classes for Parliament to allocate the necessary budget to the problem, at least not until the heat wave of 1858. As the happy advocates of sewer reform put it, the stench ‘focused the mind’ of parliamentarians on a problem that had already been carefully documented by experts for over a decade. The problem of contaminated drains, which had been drawing only limited public interest, suddenly became a matter of widespread concern, and in the ensuing five years, one of the greatest public works projects undertaken in London to that point would transform the entire way that household waste was disposed. The Great Stink was the disrupting event that made waste public, creating entirely new structures and governing bodies concerned with the flows of municipal shit.

There are several elements to this story that deserve to be underlined. On the one hand, this is very much a story of something becoming perceptible (in this case smellable), an affective disruption that produced growing public awareness of heretofore unperceived actors. The disruption completely overhauled how London would interact with water, inaugurating in the years ahead a new public that treated pipes, diseases, and odors as collective responsibilities. Modern London emerged at this moment as what Marres (2015) calls a ‘material public’ formed to address a specific breakdown in the social order’s ability to address the new collective harms of sewage. But it would be a mistake to think that all of this was simply about a breakdown, ascribing agency only to the pipes, heat, and bacteria. It was also a concerted political effort to train a new public sensibility, a work of affective reordering that allowed people to perceive the relationship between the stink, pipes, and parliament in a novel way. When the smell of the Thames reached a certain crisis point, a proposition could be made that would make sense of it, expertise could be mobilized, and solutions could be harnessed in response. Only with both the breakdown and the mobilization of a new public did the Big Stink become an event with a name, narrative coherence, and a cast of characters who continue to be remembered today.^4^

The interceptor system inaugurated in London, which segregates sewage from other forms of urban water by piping it away from the city, became the norm across Europe and eventually the colonies. This included Montreal. The city’s very incorporation, in 1834, had turned on the question of water hygiene, the building of the continent’s first covered public sewer, and the creation of a public water provision system that was the basis for municipal taxation (Dagenais, 2017; Fougères, 2004; Gagnon, 2006). By the turn of the 20th century, a series of cholera and typhoid outbreaks accelerated the production of sanitary infrastructure, progressively piping wastewater underground into a series of larger and larger ‘collector’ pipes. This piecemeal construction still relied on the St. Lawrence (a far larger river than the Thames) to disperse raw sewage toward the Atlantic. Frequent complaining about the smell of sewage in the port, from the 1890s onward, eventually led to the construction of an interceptor and water treatment system very similar to London’s, although not until the 1980s.^5^ Although all of these stages were important, none had quite the eventful quality of the Big Stink, since rather than seriously changing the way anyone thought about sewage, it simply ushered Montreal along a story of urban progress patterned by prior experiences in Europe and other North-American cities.

A catastrophic breakdown in Montreal’s interceptors, or a widespread public revolt against Montreal’s sewage treatment strategy would have been infrastructural events of huge importance in the city. Neither could have been easily dealt with through material or affective maintenance, and in addition to creating significant costs for the municipality, might have permanently changed the way Montrealers thought about the underground. But neither of these happened. Instead, some minor physical problems with the interior of one of the interceptor pipes lead to a scattershot public outcry that threatened, but never quite succeeded, in disrupting the infrastructural order of things. To study this ‘almost-event’ is to study how cities deploy affective maintenance to keep infrastructure intact.

## The Big Flush as almost-event

At the time of the Big Flush, Montreal was primed for a far more consequential reckoning with its sewage infrastructure. In the previous decade, the city had been the site of another infrastructural event with deep and lasting consequences for municipal politics. A series of scandals, both material and financial, had led to a public inquiry into municipal corruption. The Charbonneau Commission, as it was popularly known, uncovered a longstanding web of collusion in the issuance of construction contracts, between City Hall, the Montreal mafia, and some of Canada’s most prominent construction firms (see Saint-Martin, 2015). It led to the conviction not only of the sitting mayor, but also, a few months later, of the man elected to replace him, as well as the mayor of Montreal’s largest suburb. Public testimony for the commission lasted two years, and significantly changed infrastructural affect in the city: Not only did Montrealers become aware of a web of colorful criminals exchanging envelopes of cash in the backrooms of lavish clubs, they also learned about the degraded quality of cement that had been used by the city since the 1960s, and could now link these directly to the slabs falling from underpasses, or the rusted metal weave growing ominously under the city’s biggest bridges. Montreal’s infrastructural corruption had been, until that point, an ‘open secret’, a tense affective relation that nonetheless maintained a certain stasis. But a handful of egregious events brought those tensions to the surface, and the commission was charged with realigning the relationship between infrastructure and politics in the city.

The sitting mayor at the time of the Big Flush was one of the beneficiaries of this realignment. Denis Coderre, a prominent figure in federal politics, had presented himself for the mayoral election in 2013 as an outsider who could clean up City Hall. A populist technocrat, Coderre combined a larger-than-life persona with a promise of transparency, and surrounded himself with a team of respected local figures who had escaped the commission unscathed. In other words, good infrastructural management was central to Coderre’s political brand. The Big Flush occurred almost exactly two years after Coderre became mayor, and threatened to consume his office in its own infrastructural scandal.

The problem was this: The interceptor needed to be closed for repairs. But closing the interceptor meant that the raw sewage it carried to the treatment plant would have to go directly into the St. Lawrence River. As per standard maintenance procedure, the City had studied the matter and decided that the only people who would be truly inconvenienced were a small group of kayakers and river surfers who braved the treacherous rapids just below the various sewage outflows. So after posting an announcement of the work on the website dedicated to it, they also put out a quiet press-release that offered a bland proposition about the Flush: ‘the people most concerned will no doubt be athletes surfing in the Sault Normand rapids’ (Blais, 2015). Beyond merely complying with the law by posting the maintenance plan, the City was anticipating the harms that the plan might cause and the people who might be affected by them. The announcement about the repairs was also, therefore, an attempt at interpretive containment, at acknowledging impact while restricting how much of a new material public might form out of concern for those impacts.^6^

In this light it’s also easy to see what the opposition party was trying to do when it released a proposition of its own, trying to reframe the closure of the sewer line. Known for its social democratic and environmentalist leanings, Projet Montréal published its statement the next day, questioning whether the administration had the competence or will to find alternatives to a full flush.^7^ According to them, the harm, or risk of harm, extended far beyond surfers to all people and creatures using the river around Montreal and downriver. Amplified by local newspapers, Projet Montréal’s statement of concern quickly found an audience. One article, titled ‘Eight Billion Liters of Wastewater Will Be Spilled into the River in Montreal’, provided the sordid details that would become widely known: ‘Thirteen cubic meters per second during seven days, that’s eight million cubic meters or eight billion liters, the equivalent of 2,600 Olympic swimming pools of toilet refuse, hospital and commercial waste. A festival of bacteria, viruses, and pharmaceutical products directly in the river, without going through the purification plant’ (Gerbet, 2015). The City’s spokesperson was sent out to reassure the public that the impressive flow of the St. Lawrence River — ‘6,000 to 7000 cubic meters per second’ — would make the spill inconsequential for the environment.^8^ But they had clearly failed to contain the story, which now threatened to become a major controversy.

Concern over the Flush very quickly extended beyond Montreal itself. Given the flow of the river, the downstream receivers of the effluent were even more affronted than those whose toilets would be feeding it. The mayor of Sorel-Tracy, a town just downstream, expressed his discontent by saying, ‘We are not a draining field for the City of Montreal’ (CBC, 2015c). Odanak and Wôlinak Abenaki communities also expressed their opposition (Agence QMI, 2015). If, for the City of Montreal, October was the best time of the year to conduct its operation,^9^ for Abenakis the Flush would coincide with traditional fishing activities in Lake Saint-Pierre, where the river widens downstream of Sorel. For Odanak Abenaki councilor Alexis Wawanoloath, the spill ‘could constitute a serious hindrance to Abenakis traditional activities and ancestral and treaty rights’ (Agence QMI, 2015). Wôlinak chief Denis Landry, for his part, wrote to federal and provincial environment ministers, demanding to be consulted on the project before any kind of authorization was given (Courrier Sud, 2015).

The news even made international headlines (CBC, 2015f). New York State Senator Patty Ritchie declared herself ‘extremely disappointed’ that the City of Montreal had neglected its infrastructure to the point of having to soil this ‘national treasure’. The Senator also expressed worry that Montreal, ‘which has been known as one of the world’s most environmentally friendly and clean cities’, would be sending a message to other riparian municipalities that the river could henceforth serve as ‘dumping ground for sewage and other dangerous pollutants’ (Ritchie, 2015b). Senator Ritchie even appealed to the International Joint Commission, the bi-national authority for transborder waters, to investigate the municipal decision (Ritchie, 2015a). All of these complainants eventually resigned themselves to the inevitability of the plan, but not without objections that they had either not been fully informed, or that they ought to have some right of authorization over such circumstances (Rochette, 2015a, 2015b).

All of the actors in this story were doing different kinds of affective work, trying to create publics with particular reactions to infrastructural conditions. Even though they appealed to legal structures, to authority and authorization, all of the referents for these appeals were themselves open to contestation. In fact, the St. Lawrence river is a notorious site of jurisdictional overlap: while provincial governments have broad powers and ownership over water resources within their territories, the federal government has jurisdiction over fisheries, navigation, and shipping (Doelle & Tollefson, 2013, pp. 166–172; Dorion & Lacasse, 2011). Even the figure of ‘the environment’, which was most often named as the victim of the Flush, does not have a stable legal position. The environment does not figure in the Canadian constitution but began showing up in federal court cases in the 1980s, with marine pollution quickly recognized as falling under federal jurisdiction (Doelle & Tollefson, 2013, p. 173). But in Canada federal power itself is highly contested (see Cameron & Simeon, 2002; Gagnon, 2010; Mackay, 2013). Although there are cases where different levels of government are keen to claim authority over environmental questions – for example, over contentious oil pipelines or fracking permits – the questions of who deals with contamination often leads to governments ‘passing the buck’ to each other (Harrison, 1996).

In federal law, a deliberate dumping of ‘deleterious substances’ into the river would contravene section 37 of the Fisheries Act, and thus require approval under its 2012 *Wastewater Systems Effluent Regulations*. These regulations were meant to ‘set the national baseline quality standards for effluent discharged from wastewater facilities’ (Miller Thomson LLP, 2013). However, to avoid regulatory duplication, the new legislation allowed equivalence agreements with provinces having provisions that are ‘deemed equivalent’ (Canada, 2018). In 2014, the Government of Quebec enacted the *Regulation Respecting Wastewater Treatment Works*, which allows wastewater dumps during maintenance (see Gouvernement du Québec, 2019), and requested an equivalence agreement in March 2015, which had not yet come into force at the time of the Flush.

All of this overlap and ambiguity about jurisdiction created ways to move questions around, reframe them, and claim or deny responsibility for them as a way to manage the affect surrounding an impending event. The play between them was also significantly affected by a federal election planned to happen around the same time as the Flush. On October 7, federal Environment Minister Leona Aglukkaq tweeted that, ‘Last week my office learned of Montreal’s plan to dump billions of litres of raw sewage into the St. Lawrence. This plan is concerning and we have done the responsible thing by exploring options to prevent it while we get more information’ (The Canadian Press, 2015). The responsible thing referred to here was a federal order demanding that Montreal suspend its plans for a week while her department investigated alternatives. After a week, Aglukkaq’s office ordered the Flush suspended again, and called for an ‘independent expert scientific review’ (Environment Canada, 2015). As we will see below, there are many layers to this invocation of expertise, but its immediate effect was deferral, allowing Aglukkaq to avoid making any specific pronouncements on the Flush until after the election.

The review bought time, time for the media to gather more opinions, for public outrage to dilute, and for the political stakes of the event to subside. The panel of experts returned with an opinion saying that nothing untoward had happened, that the closure was indeed necessary and the impact on the river would be minimal. After Aglukkaq’s party lost the federal election, the new environment minister gave the Flush a green light, adding the condition that water quality needed to be monitored for the duration of the repairs. Event averted; the Big Flush remained merely routine maintenance, part of the infrastructure itself. As the mayor was fond of repeating: ‘We’ve done this before, and we’ll do this again.’ In the cosmopolitical tumble, it was the City’s initial proposition that ultimately won out.

## Populist redistribution

The story of the Big Flush ended up playing out almost exactly as anticipated by the City, with no significant reconfigurations. The City’s success has less to do with the ways that maintenance was carried out on pipes to avoid a harmful overflow of sewage as it has to do with how various levels of government managed to avoid a growing public disruption to their authority over those pipes. We’ve been arguing that it’s worth considering such strategies as a kind of affective maintenance, since they work to avoid infrastructural events by reorganizing the relations in which they are embedded. We’ll consider two such strategies, the populist and the technocratic, both of which are often characterized as ‘anti-political’ because they dissuade the emergence of contestation (Swyngedouw, 2011). To be clear, this needn’t be thought of only as critique: Cities would be unliveable if sewers were constantly eventful, and one of the things we expect of responsible government is this sort of maintenance. But it is also helpful to remember that uneventfulness serves the political interests of some and works against others. Affective maintenance is a kind of minimally disruptive reordering of infrastructural relations, not strictly apolitical, but a kind of politics oriented toward maintaining the status quo.

The most obvious ploy to rearrange infrastructural affect in his favor was made by Mayor Coderre, who grew increasingly frustrated with public outcry over the Flush and began to disqualify the propositions of his opponents for being ‘political’. Reacting to the suspension of work by the federal environment ministry, he claimed that none of this had anything to do with infrastructure or the environment, but rather with political brinksmanship. ‘They have this file in their hands [since September 2014] … We asked, ‘Do you have more questions? Do you have more questions?’ They had no questions. … We don’t need to play politics on the back of Montrealers’ (CBC, 2015d).^10^ If there was a threat facing Montrealers, it was not from untreated sewage, but from politics itself. As a rhetorical strategy this is a classic populist one: Coderre defined a homogeneous subject (Montrealers) whose interests were threatened by division from outsiders (Swyngedouw, 2007).

As Laclau (2005) has pointed out, populism works by calling into being a certain kind of public, the ‘people’, whose interests are universal and therefore transcend the bickering associated with politics. For the Coderre administration, infrastructural populism took a very particular form, maturing into a strategy for linking Montrealers’ newfound interest in the pipes themselves. On its website, the City published an information page about the Flush suggesting that wastewater was ‘a collective responsibility’, and citing ‘some things that you must not flush down the toilet, now or ever: wet wipes for babies or personal hygiene; diapers; Q-Tips; hair; dental floss; tampons; condoms; expired medication; cooking grease’ (Ville de Montréal, n.d.). In other words, it invited a public newly aware of the interceptor’s existence to extend their awareness further, to consider how the pipe was connected to their most personal quotidian practices. It directly mobilized citizens for infrastructural service (Carse, 2012; Loftus & March, 2016) by asking them to provide part of the filtration normally associated with the purification plant. Coderre, in appealing to Montrealers’ intimate relationship with sewage, also turned the gaze on himself, performing an ‘everyman’ stunt by leaving his office and descending into the sewer accompanied by journalists. Perhaps the most enduring image of the Big Flush is that of Coderre donning a yellow Hazmat suit and climbing down into a manhole under the street (Pineda, 2015). The group named ‘Montrealers’ here all became honorary members of the maintenance crews carrying out the Big Flush, and indeed the Big Flush itself became the sum of many little flushes.

Of course, populist gestures also contain traps for leaders who invoke them. If everyone is part of the sewer, then why do we have a City Hall? The public responses to this call for individual responsibility suggested that few Montrealers were interested in joining this particular infrastructural public. Richard Fontaine, Montreal’s director of wastewater treatment, suggested a ‘simple test’ by which citizens could measure their contribution to the river’s health: ‘before you throw it in the toilet bowl, would you put it in your pool or your bathtub?’ (Bruemmer, 2015). The slightly disingenuous suggestion invited a raft of mockery, with at least one angry comment on the story suggesting that to protest the Big Flush citizens ought to ‘Put the sewage in Denis Coderre’s bathtub.’^11^ If a populist approach to maintenance was to invite a homogeneous public to identify themselves with the city’s infrastructure, these comments offered a simple carnivalesque inversion that refused the administration’s condescension and recalled the centralized hierarchy of municipal politics. What all of this reminds us is that, just like the maintenance carried out on pipes, maintenance of public discourse involves a certain amount of improvisation and contingency, as well as dangerous moments of slippage (Denis & Pontille, 2019).

## Expertise as maintenance

If populist maintenance didn’t work all that well, the set of interventions that were most successful were the appeals to expert knowledge. Rendering a problem technical can be a strong form of disqualification that works by closing the parameters of argument around a rarefied discursive community authorized to speak about ‘science’ (Stengers, 2010). In effect, expertise seeks to make certain kinds of propositions inadmissible. What the various invocations of expertise showed in this case was that there is an even simpler function for expert knowledge in affective maintenance: It can act as a form of deferral and delay. Thus, the public that was emerging around the anticipation of the Flush could be told to hold off while experts studied the dilutive capacity of the river once more, the likely effects on specific marine inhabitants, and downriver water quality. It could also be deferred by the mere production of information, on the promise that careful study, whatever its conclusions, would eventually lead to clearer representations of a problem, and that clarity of representation would lead to clarity of decision (Hetherington, 2012). Calling on expertise brings with it certain political risks, but these risks might be worth its effectiveness at buying time.

In the case of the Big Flush, the emptiness of expertise became evident when the federal environment minister convoked an advisory panel of experts to study the case. As Sarah Dorner, an independent observer of the process, remarked at the time, the St. Lawrence river ‘is one of the most well-studied in the world and some of the best expertise in this area is actually housed within Environment Canada and so it is surprising that they would need an independent expert’ (CBC, 2015a). Furthermore, she noted that there was very little legal support for such a panel, and that the federal government had no jurisdiction to act on its recommendations anyway.^12^ If the federal government had attempted to block the Flush, they would probably have been unsuccessful. But the request for a panel review was less drastic and gave everyone a chance to agree without conceding anything but time.

The actual mandate given to the independent scientists was to evaluate potential impacts.^13^ But even if the expert panel had no authority to approve, the federal government created the public impression that scientists approved of the Flush. Water management experts whom we interviewed after the case were clear that both the decision to study the Flush, and the ultimate decision to Flush, were political moves ‘not based on any kind of scientific opinion’.^14^ And yet these experts found that responsibility for the Flush had been attributed to them anyway. Pressure on the three panel members was so great that they eventually decided to sever all contact with the media to avoid constantly having to defend their competence or to deny that the Flush was their idea. As one of the three told us, ‘I would meet someone that I know and [they would] say, “Oh, okay, I saw that you were on the panel, it’s so sad that you recommended that discharge.” And then I would have to explain, “No, I wasn’t asked to make a decision.”’ Even if the panel had no real authority, its creation served to promote a perception of a ‘rule of experts’ (Mitchell, 2002).

The public misperception that the expert panel had any power in the process was all the more galling to its members because they themselves were ambivalent about the Flush. Hydrologists and engineers we talked with about the Flush all said they thought it was good to see Montrealers protesting what they saw as a woefully inadequate treatment system. And yet our interviewees often gave a chuckle or eye-roll when talking about it and said that the circumstances surrounding the Big Flush could never have addressed the ‘real’ problems of Montreal’s sewage infrastructure. One of them, whom we’ll call Sylvie Lapointe, evoked it metaphorically: If public pressure was positive, at the infrastructural scale its distribution was too concentrated and dissipated precisely at that moment when it could have been applied constructively, namely *after* the crisis and in a more sustained way. For her, the dumping of untreated effluent into the river was all too ordinary. Two spills in 2003 had sent a total of 17.6 million liters into the St. Lawrence, while another in 2005 had spilled 770 million liters (CBC, 2015c). Wastewater also overflowed into the river whenever heavy rains would overwhelm the city’s sewer network, which only required about 10 mm of rain. For critics who knew the sewer system in more detail, the scandal was not so much the single Flush itself, but the recurrent flushes of an inadequate system. In the province of Quebec as a whole, accounting for every municipal sewer event, water advocates counted 62,000 sewage spills in 2017 (Eau Secours et al., 2018). Knowing this, no panel of experts could get exercised over a planned spill associated with maintenance. The horror of Montreal’s water treatment system was its banal, everyday inadequacy, a form of slow (uneventful) violence that almost never elicited any public scrutiny (Nixon, 2011).

For different actors, in other words, there were different timescales at work in how people perceived the Flush and the sets of relations it entailed, and experts convoked to comment were caught in a double bind. On the one hand, they believed that the public outrage surrounding the Flush needed to be adjusted to the deeper time of infrastructural change, an opportunity for citizens to get to know their sewers more intimately and learn to critique the City on its long-term strategy for dealing with wastewater. And yet, slowing down public discourse to better understand the technical complexity of a problem also had the effect of dissipating that outrage and losing its potency. The experts who lamented the redundancy of the study speculated that the panel had been created as a diversion from the deeper questions about municipal plumbing. Explaining her ideal response to the Flush, Lapointe invoked a different sort of citizenship, one less moved by sudden bursts of outrage and more attuned to the temporality of infrastructure, and more cognizant of the slow infrastructural violence of long-term neglect. In other words, Lapointe also longed for the emergence of a new public, but it was a public that would be much less affective – a public made up of experts like herself who could dispassionately review information.

In a way, Lapointe is demonstrating precisely why expertise is good for managing affect. Because even while she disagrees with the City, she agrees that management is better achieved when one removes strong feelings from the equation. But as the story of the Big Stink so aptly shows, infrastructures rarely change until they become eventful, and their eventfulness is by definition an affective shift. Arguably, it is in these moments of affective rupture that these forms of representative democracy are at their most interesting, as voices, circumstances, things, and events erupt onto deliberative processes from which they have otherwise been excluded. This was always the potential of the Big Flush, that it might become eventful, to break through and provoke a systemic realignment, a structural rethink of how a city treats its refuse. But for that to have happened, expert critics of the city’s sewage infrastructure would have had to see themselves aligned with those calling to stop maintenance work or dump sewage in the mayor’s bathtub, even if those opinions spoke a different language and seemed to operate at a different timescale. Instead, they found themselves called to shore up the government’s position, not because they agreed with all of its implications, but because it was more technically accurate. In doing so, they ended up participating in the closure of a debate that they thought was healthy.

More expertise, while certainly vital in producing knowledge about the environment, can also end up promoting inaction.^15^ In Quebec over the past decades a series of administrative innovations have grown up around water, including not only the jurisdictional vagueness evoked earlier, but also fine-grained deliberative bodies designed on the principle of integrated management and the concept of *concertation*.^16^ These measures have contributed to the proliferation of jurisdictional, territorial, and institutional forms whose relation to pre-existing decision-making infrastructures remains ambiguous at best (Milot, 2009). Like the independent experts consulted at the height of the Big Flush crisis, the authority of these bodies is tenuous (see Schmidt, 2017) though their processual activities do participate in a kind of affective maintenance of administrative relations to water in the province.

It is interesting to note that while Coderre argued that his municipal government had done its due diligence by involving the relevant provincial and federal authorities in a timely way, none of these integrated water management bodies had in fact been involved in the process. When we talked with one of their administrators about this, she argued that the controversy around the Big Flush had been mostly due to a failure of communication. Had the City used the para-infrastructure designed in part for this purpose, they might have been able to smooth over the gap between popular imagination and the imperatives of government.^17^ Yet one could also argue that the proliferation of redundant bureaucracies without clear governing mandates is one of the ways that the rule of experts works. If the function of such bodies is less to constitute a decentralized political apparatus than to contain affective overflows by pushing the conversation into a more dispassionate realm, then it is not surprising that they occasionally get neglected and overlooked, or supplemented by yet more redundant forms of expertise.

## Conclusion

The Big Flush turned out, in the end, to simply be a well-planned plumbing job. On November 11, at one minute past midnight, Montreal shut off the interceptor’s valves. The complete shut-off lasted only 70 hours, and the interceptor was put back into full operation on November 14, at 10 pm.^18^ In its report, the City recorded that, ‘The effluent’s plume was visible and odors were detected only in a few locations’, and ‘that the discharge of used waters did not cause measurable medium- and long-term effects on the receiving environment’ (Ville de Montréal, 2017). More importantly, the controversy also dissipated, and with it much chance that the Big Flush would reveal anything wrong with Montreal’s sewage infrastructure. It was a non-event, and the pipe continued to function as it always had, beyond most Montrealers’ awareness or concern. In the cosmopolitical scheme of things, then, it was City Hall’s proposition that carried the day, and criticism of the Flush could now be disqualified as ill-informed or politically motivated, or both.

We’ve suggested that the work that went into producing, maintaining, and restoring this uneventfulness is indicative of a larger, generally unappreciated cosmopolitical labor behind modern infrastructure in democratic societies. More than simply physical reliability, the uneventfulness of infrastructure is produced by affective maintenance. Those practices include disqualifications of counterarguments, populist appeals that re-orient emergent publics, and deferrals and delegations to expert knowledge. This is vital work. When true infrastructural events take place, they call new publics into being which threaten to fundamentally reinterpret what infrastructure is for, what standards ought to govern it, and who ought to be responsible for the technical order underpinning modern life.

We return in the end to Sylvie Lapointe’s hope that a more expert form of citizen might emerge to better appreciate sewage and sustain a critical relation to its management, in order to suggest another possible mode of expert involvement. If most of the experts in this story were unhappy with the way their expertise was deployed, it was in part because they accepted a narrow definition of their role: to offer authoritative statements of fact based on constrained variables. Under those conditions, they found themselves not only in agreement with the City’s proposition about the Flush, but complicit in disqualifying other opinions about the larger question of how cities manage their sewage. The catch-22 in which they found themselves hints at another way of being expert, that resists being taken up in the redundant forms of discursive maintenance and instead embraces the multiplicity of propositions that infrastructural events can create. A more cosmopolitically savvy expertise might find ways to align with emergent material publics, even when these seem hyperbolic or misinformed. Only through them and with them might they find ways to make political the slow violence and routine inadequacies of the status quo.

## Notes

1. Research was carried out as part of the Montreal Waterways Project, an ongoing collaborative project of students at the Concordia Ethnography Lab. In addition to the two authors of the paper, Alix Johnson, Tristan Biehn, and Alejandra Melian-Morse were key to gathering the materials and interviews for this paper. The team carried out three interviews specifically on the Flush, but it often came up as a topic in interviews carried out on other aspects of Montreal’s waterways. For more on the larger project, see: https://ethnographylabconcordia.ca/working-groups/montreal-waterways/

2. This is a slightly different understanding of infrastructural events than that laid out by Carse (2017) as an event that, due to its infrastructural distribution, precipitates unexpected consequences across time and space. Our own understanding of ‘events’ is informed by Wagner-Pacifici’s (2017) discussion of events as narrative cleavages.

3. This is part of what Michael (2020) calls ‘affective infrastructuring’.

4. Lord Edwin Chadwick, the public health pioneer, and Joseph Bazalgette, the engineer, usually appear alongside prime-minister Benjamin Disraeli and Queen Victoria in this tale.

5. This was extremely late. The dilutionary capacity of the St-Laurence is truly remarkable, and allowed Montreal to avoid building an interceptor until a century after many US cities. Even Toronto, which was smaller than Montreal until the 1970s, built its own interceptor in 1913 (Benedickson, 2007). Indeed, the Montreal station was built so late that by the time it was built it had to be gigantic. Unlike other cities, which built small stations as their cities grew, Montreal built a single station which for many years was the worlds largest.

6. This is at least a tacit acknowledgment of the truth of John Dewey’s understanding of publics, as outlined by Marres (2015). According to Dewey, publics form around particular sets of material relations that cause collective harms. The announcement by the City suggests a form of pre-emptive work on public-making: Acknowledging that the Flush might harm some surfers, it also suggested that this harm was minimal. By naming the harm as affecting a very small community of relatively privileged people, it also headed off the possibility of widespread identification or sympathy.

7. Projet Montréal’s press release from September 28, 2015, can no longer be found on the party’s website, as for other publications from this period. When the party won municipal elections in 2016, they seem to have erased the record of their stance during this period.

8. The apparent self-evidence of these numbers notwithstanding, arguments over dilution are a cornerstone of the history of sewage treatment, and as Benedickson (2007, p. 179) points out, engineers had been having these argument about Montreal since at least 1909.

9. Montreal Mayor Denis Coderre, in a letter dated October 16, 2015 and addressed to Federal Environment Minister Leona Aglukkaq, described this time period as ‘the period of the year that offers the most favorable hydraulic and environmental conditions, which precede snow removal operations and during which impacts on fish and river users will be minimized, that is, between October 15 and November 15, 2015’ (Coderre, 2015).

10. Coderre’s retort was corroborated by the Canadian Press, who found documents revealing that the City had done its diligence with the province for over a year, and that the Federal Environment Ministry had confirmed knowledge of the dossier since at least September 2014 (The Canadian Press, 2015).The provincial authorization certificate was delivered on February 27, 2015 (Ville de Montréal, 2017).

11. The comment was made by Hank Cookie Rich in the comment section of a CBC article (CBC, 2015e).

12. As Mayor Coderre noted in a letter to the Minister: ‘Given the existence of a regulatory framework covering the temporary diverting of untreated wastewater, we are of the opinion that the issuing of an interim order under Section 37(2) of the Fisheries Act is inappropriate and abusive in the dossier’s present circumstances and that issuing an interim order in the context of a temporary diverting of wastewater respects neither the spirit nor the letter of the regulation applicable to the discharge of untreated sewage by municipal wastewater systems in Canada’ (Coderre, 2015).

13. Information about the expert committee comes from an interview with one of its experts, on February 14, 2018.

14. The quote comes from a hydrology professor, and we heard much the same from one of the assistant directors of a ZIP committee that would have been affected.

15. All of this is akin to the governmental strategy of calling a commission to study issues rather than dealing with them directly, and in environmental planning can easily lead to what Bavington (2011) calls ‘managed annihilation’ whereby expert management becomes the raison-d’etre for a lack of environmental caution or care.

16. One of these is the provincial-federal framework St. Lawrence Action Plan, initiated in 1988. The other is the provincial watershed administration scheme stemming from the 2002 National Water Policy. For an overview of these innovations in hydrosocial management on a global scale, see Schmidt (2017).

17. Interviewed on March 23, 2018.

18. In total, the work lasted for 89 hours and a total of 4.8 billion liters were discharged, so 3.2 billion less than initially estimated, over a much shorter period.
